# Bumbledom at Birmingham

**Published:** 1889-10-12

**Authors:** 


### The Hospital

A Weekly Institutional Journal of

Science, Medicine, Nursing, and Philanthropy.

*For Hospitals, Asylums, Medical Practitioners, Students, Nurses, Families, Parishes, Congregations, and the Charitable Public.*

Vol. VII. No. 159.] [October 12, 1889.

### BUMBLEDOM AT BIRMINGHAM.

A fresh chapter in the history of sanitary progress has been written and completed by the passing of the Act for the Compulsory Notification of Infectious Diseases. Doctors who have humanity enough to identify themselves with their fellow men in their daily needs and cares, have striven long and arduously for the passing of a measure of this kind. If there has now and then been sounded a note of opposition to the Bill in its progress, it has not been opposition to the main principle of the measure, but only to certain proposed details which threatened to put responsibility on the wrong shoulders. The Act in its present form, however, and as a tentative measure, undoubtedly satisfies all reasonable demands, and, therefore, justly claims the confidence and loyal support of the public, both lay and medical.

It may be desirable to explain to lay readers that the object of the Notification of Infectious Diseases Act is to bring to the immediate knowledge of local sanitary authorities any and every case of infectious disease which may arise; and the purpose for which this knowledge is required is that every such case shall be at once and thoroughly isolated, either in the house where the outbreak has occurred, when that is practicable, or in a public building provided for the purpose if private isolation cannot be secured. It is proved beyond possibility of doubt that complete isolation of this kind rapidly stamps out infectious disease; it is demonstrated with equal certainty that in no other way can an epidemic by any possibility be arrested. The "Notification of Infectious Diseases Act" might therefore have been called with equal, or even greater, propriety the "Protection of the Public against Infectious Diseases Act," for that is what it really amounts to in practice.

Such being the facts, it is natural to suppose that all local sanitary authorities will promptly place themselves under the provisions of the Act, which, by the way, is permissive to that extent; and that having chosen to place themselves under its provisions, they will see them thoroughly carried out. For of what use is the Act unless it be applied thoroughly, both in the spirit and in the letter? The chief duties, of course, that devolve upon the local sanitary authorities are two: first, the provision of adequate hospital accommodation for infectious diseases; and, secondly, the effective supervision of the sanitary area committed to their charge. All this would seem quite easy of accomplishment, since the local sanitary authorities, having control of the rates, have nothing to do but to order provision to be made, and the thing is at once done.

Such is the simplicity of the line of duty in theory. What is the performance in actual practice? Birmingham may be taken as a fairly representative English municipality. It has always prided itself on being a thoroughly progressive and business-like town; nay, it has, for several decades at any rate, claimed the place of leader on questions of high public and imperial policy. Where, then, does Birmingham stand in relation to the new departure which has for its object the stamping out of dangerous diseases and the saving of human life? Is she in the forefront, showing other municipalities how promptly, smoothly, and effectively she can carry out a great measure of public utility, or is she like a belated rural district far in the rear? A correspondence on the subject, which has recently been carried on in the *Birmingham Daily Post*, demonstrates the painful parochiality of the average doctor and municipal ruler of Birmingham. The controversialists were Dr. Bostock Hill, who holds the diploma in public health of the University of Cambridge, and Mr. Edwin Chesshire, a Fellow of the Royal College of Surgeons of England; and these gentlemen have displayed a zeal in the splitting of hairs, and in repeated attempts to disparage one another without quite descending to the level of the stable-yard, which, if expended on the instructing of the public mind in the provisions and utility of the new Act, would have rendered a public service which would have been invaluable.

Thus far the doctors! What the municipal rulers are doing to carry out the beneficent enactments of the measure may be seen from a letter which appeared in the *Post* of September 16, signed "W. D." and which we here quote. Says "W. D.": "Without any desire to hamper the action of the authorities, who seem to be"—mark "the seem to be"—"acting energetically in the face of great difficulties, it may be as well in the interests of the public to note the following: As a medical man in temporary charge of a practice I was called in last Tuesday to a case of scarlatina. The rash was fading, and there were four other children in the house, all of whom had been freely exposed to the risk of infection. There were two beds only, and the mother, a poor woman, had no means of keeping the invalid apart, or of sending the rest of the family away. Under the circumstances it seemed to me best to leave the boy on the couch in the living-room, where a bed had been made up, and to get him away to the hospital as soon as possible. The same night I sent in an official notice, and the inspector called next morning. The mother tells me he said there were twenty cases waiting admission, but the child would be taken in whenever there was room. Nothing further has been heard by the mother, and the child is now going through the desquamative stage, which, as most people know, is the most infectious period of the disease. Under these circumstances you will perhaps excuse my saying that it would convey a more accurate view of the range of the epidemic if the reporter of the *Post* were to make strict enquiries, not only as to the number of admissions, but also as to the applications that have been shelved through want of accommodation."

There is a severe epidemic of scarlet fever raging in Birmingham, and this is the way in which the local sanitary authorities meet the emergency. The object of an infectious hospital is isolation, and the reduction of centres of infection from scores or even hundreds to one. Here, if "W. D." reports correctly, were twenty cases, producing twenty centres of infection, all left to spread the poison of a dangerous disease through a crowded population, because the local rulers had neglected to provide sufficient means of isolation. It may be said that the Compulsory Notification Act is new, and that Birmingham has only recently adopted it, or has not adopted it at all. But that is the poorest possible pretence at an excuse which careless and imcompetent sanitary authorities could think of making. The real points are these: Do the sanitary authorities of the Midland capital know that isolation is imperative for the stamping out of infectious diseases; and that of all infectious diseases scarlatina is one of the most virulent? Did they not know this long before a Compulsory Notification Act was ever thought of? Knowing it thoroughly and well for years, was it not their duty to have provided sufficient means of isolation long ago? What is the great plea for local self-government which receives such strenuous support in Birmingham? Is it not that populous localities understand and can manage their own local affairs better than those affairs can be managed from a distance? A perfectly valid plea, we admit, but then we expect to see it justified in practice; and of all places in the world, at Birmingham. Yet here we have a complete, miserable, and dangerous failure of local self-government at the very heart and centre of local self-government principles; and the only conceivable excuse is that the local self-governors were not instructed, driven, and compelled to do their duty by the Imperial authorities at Whitehall. Is it possible to conceive a more abject and discreditable condition of affairs?

We have quoted this case, and tried to picture it in its true light, because it well illustrates the state of things which is extremely prevalent in both large provincial towns and rural districts. The way of sanitary salvation is open, but whole populations, with their local leaders, stubbornly refuse to walk in it. What are the causes of their want of sanitary faith? Ignorance and greed! Nothing else! Ignorance, which is mentally incapable of seeing and understanding the public and private value of scientific sanitation; greed, that has not the breadth and self-preserving liberality of mind to dare to add a single penny in the pound to the rates. And this is local self-government in actual operation! There is not a word to be said against local self-government in principle. It is, on the contrary, an imperative necessity of the times, at any rate in this country. But it is impossible to feel other than ashamed of our fellow-countrymen when they show so complete an incapacity to rise above the narrow limits of a petty parochialism, and to deal with the necessities of a progressive age in a broad, manly, and progressive spirit. If Birmingham would retain her reputation as a provincial leader, she must promptly rid herself of counsellors who cannot guide, and do with all her might the plain and obvious duties of the day.

